# Gamma-Tocotrienol Induces Apoptosis in Prostate Cancer Cells by Targeting the Ang-1/Tie-2 Signalling Pathway

**DOI:** 10.3390/ijms20051164

**Published:** 2019-03-07

**Authors:** Kai Dun Tang, Ji Liu, Pamela J. Russell, Judith A. Clements, Ming-Tat Ling

**Affiliations:** The School of Biomedical Sciences, Australian Prostate Cancer Research Centre-Queensland & Institute of Health and Biomedical Innovation, Queensland University of Technology and The Translational Research Institute, Queensland 4102, Australia; Ji.Liu@qimrberghofer.edu.au (J.L.); pamela.russell@qut.edu.au (P.J.R.); j.clements@qut.edu.au (J.A.C.)

**Keywords:** prostate cancer, angiopoietin-1, Tie-2, gamma-tocotrienol and autophagy

## Abstract

Emerging evidence suggests that gamma-tocotrienol (γ-T3), a vitamin E isomer, has potent anti-cancer properties against a wide-range of cancers. γ-T3 not only inhibited the growth and survival of cancer cells in vitro, but also suppressed angiogenesis and tumour metastasis under in vivo conditions. Recently, γ-T3 was found to target cancer stem cells (CSCs), leading to suppression of tumour formation and chemosensitisation. Despite its promising anti-cancer potential, the exact mechanisms responsible for the effects of γ-T3 are still largely unknown. Here, we report the identification of Ang-1 (Angiopoietin-1)/Tie-2 as a novel γ-T3 downstream target. In prostate cancer cells, γ-T3 treatment leads to the suppression of Ang-1 at both the mRNA transcript and protein levels. Supplementing the cells with Ang-1 was found to protect them against the anti-CSC effect of γ-T3. Intriguingly, inactivation of Tie-2, a member receptor that mediates the effect of Ang-1, was found to significantly enhance the cytotoxic effect of γ-T3 through activation of AMP-activated protein kinase (AMPK) and subsequent interruption of autophagy. Our results highlighted the therapeutic potential of using γ-T3 in combination with a Tie-2 inhibitor to treat advanced prostate cancer.

## 1. Introduction

Prostate cancer is one of the most commonly diagnosed cancers in men worldwide. Although most of the newly diagnosed cases are at low risk [1], many patients still suffer from disease progression due to the development of metastasis, which is the major contribution to the morbidity and mortality of the disease [2,3]. At this stage, chemotherapy and radiotherapy exhibit only limited benefits. Therefore, advanced prostate cancer remains incurable with current treatment strategies.

Tocotrienols (T3s) are members of the vitamin E family and have been shown to possess anti-cancer properties [4,5,6,7]. As reported by Srivastava et al. [8], a tocotrienol-rich fraction (TRF) was able to inhibit the growth and induce apoptosis of human prostate cancer cell lines (LNCaP, DU145, PC-3), but not in normal human prostate epithelial cells, suggesting that it specifically targets cancer cells. Meanwhile, we demonstrated recently that the gamma-T3 (γ-T3) isomer significantly inhibited prostate cancer stem cell (CSC) self-renewal in vitro and in in vivo prostate tumour formation studies [9]. Furthermore, we found that γ-T3 can also sensitize prostate cancer cells to the chemotherapeutic drug, docetaxel [10]. Nevertheless, the underlying mechanism responsible for its anti-cancer effect is still far from clear.

As reported by Yap et al. [10], the anticancer effects of γ-T3 have been found to link with the suppression of the nuclear factor-kapppa B (NF-κB) signalling pathway. NF-κB plays an important role in regulating cell survival through the induction of a series of anti-apoptotic proteins in cancer cells. Moreover, γ-T3-induced apoptosis was also found to be associated with the suppression of NF-κB, epidermal growth factor receptor (EGFR), and Id family proteins (Id1 and Id3) in prostate cancer [10]. Besides that, Campbell et al. [11] found that the inhibitory effect of γ-T3 on prostate cancer cell proliferation is acting through the TGFβ2 pathway.

Furthermore, γ-T3 was also found to suppress PI3/AKT activation, which is crucial for maintaining the survival, proliferation, and invasion of cancer cells [12]. Although γ-T3 has a limited effect on the activation of the AMP-activated protein kinase (AMPK) pathway, our recent study suggested that it can act synergistically with other natural anti-cancer compounds to activate the AMPK signalling pathway. As demonstrated, both in cell lines and a xenograft model, treatment of prostate cancer cells with both T3 and the anti-cancer compound, polysaccharopeptide (PSP), was found to activate AMPK [13].

Apart from directly inhibiting the growth and survival of cancer cells, T3s have also been shown to suppress tumour angiogenesis [14,15]. Inokuchi et al. [16] was the first group to report that T3 inhibits angiogenesis by suppressing the proliferation and tube formation of endothelial cells. Similar findings were reinforced by Miyazawa et al. [14]: alpha, beta, and γ-T3, but not tocopherol (TP), were found to inhibit angiogenesis in in vitro and in vivo experiments, further supporting the potential of T3 as an antiangiogenic agent. More importantly, angiopoietin (Ang-1)/Tie-2 is one of the well-known signalling pathways involved in regulating the development of angiogenesis [17].

Here, we identified Ang-1/Tie-2 as a novel downstream target of γ-T3. We found in our cDNA microarray analysis that γ-T3 treatment significantly suppressed the mRNA level of Ang-1. Subsequent analysis confirmed that γ-T3 treatment not only suppressed Ang-1 gene transcription but also downregulated its protein secretion. More importantly, we found that inactivation of Tie-2 sensitizes the cells to γ-T3-induced AMPK activation, while the exogenous supplementation of Ang-1 partially rescues the cells from this effect of γ-T3. The effects of using γ-T3 and Tie-2 inhibitor together, as shown in our experiments, suggest the potential of their combined use to treat patients with advanced prostate cancer.

## 2. Results

### 2.1. γ-T3 Downregulates Ang-1 Expression in Prostate Cancer Cells

**Figure 1:**
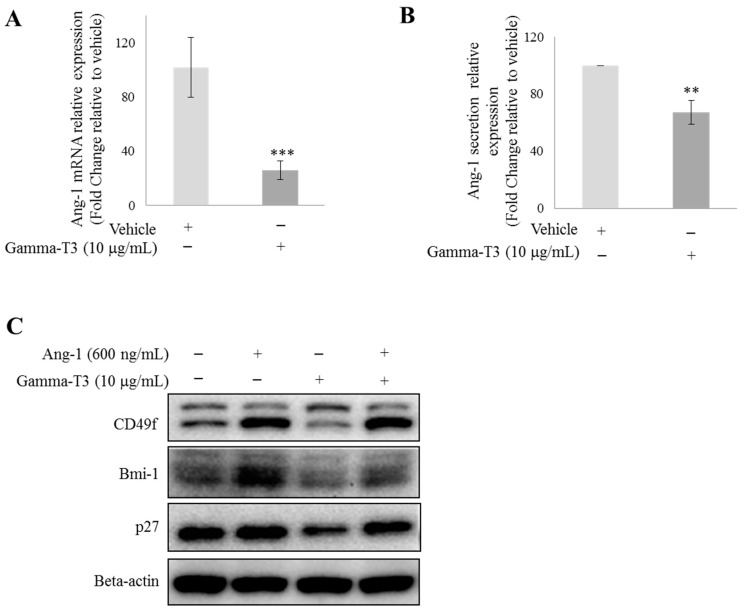
γ-T3 downregulates Ang-1 expression in prostate cancer cells. PC-3 cells were treated with 10 μg/mL γ-T3 for 72 h under a serum-free condition. (**A**) qRT-PCR and (**B**) ELISA analyses were performed to examine the effects of γ-T3 on Ang-1 expression in PC-3. (**C**) Addition of exogenous Ang-1 (600 ng/mL) partially restored the expression of CSC (CD49f and Bmi-1) and quiescence (p27) markers in the presence of γ-T3. Each experiment was repeated at least three times, and the results are presented as the mean ± SD. (*p* values: ** < 0.005; *** < 0.0005).

Recently, γ-T3 was reported to inhibit angiogenesis in both in vitro and in vivo experiments [14]. Consistent with previous studies, we found in our cDNA microarray analysis that γ-T3 significantly suppressed the mRNA level of Ang-1, a common pro-angiogenic factor in prostate cancer cells. To validate this finding, we first treated PC-3 cells with 10 µg/mL γ-T3 for 3 days under a serum-free condition [9]. γ-T3 not only significantly suppressed the mRNA level of Ang-1 in PC-3 cells, but also inhibited Ang-1 secretion from the cells (Figure 1A,B). Since Ang-1 has been shown to regulate prostate CSCs by functioning as an autocrine factor [18], we speculate that the anti-CSC function of T3 reported in our previous study may be due to the suppression of Ang-1 secretion. To test this hypothesis, we then treated PC-3 with 10 µg/mL γ-T3 with or without the supplementation of recombinant Ang-1 protein (600 µg/mL). Supplementation of exogenous Ang-1 was found to partially restore both CSC (CD49f and Bmi-1) and quiescence marker (p27) expression in γ-T3-treated prostate cancer cells (Figure 1C), suggesting that the inhibitory effect of γ-T3 on the stemness of prostate CSCs is through the suppression of the Ang-1/Tie-2 signalling pathway. Our finding, therefore, reveals that Ang-1/Tie-2 is a novel downstream target of γ-T3.

### 2.2. Synergistic Effect of γ-T3 and Tie-2 Inhibitor on Prostate Cancer Cell Growth

**Figure 2:**
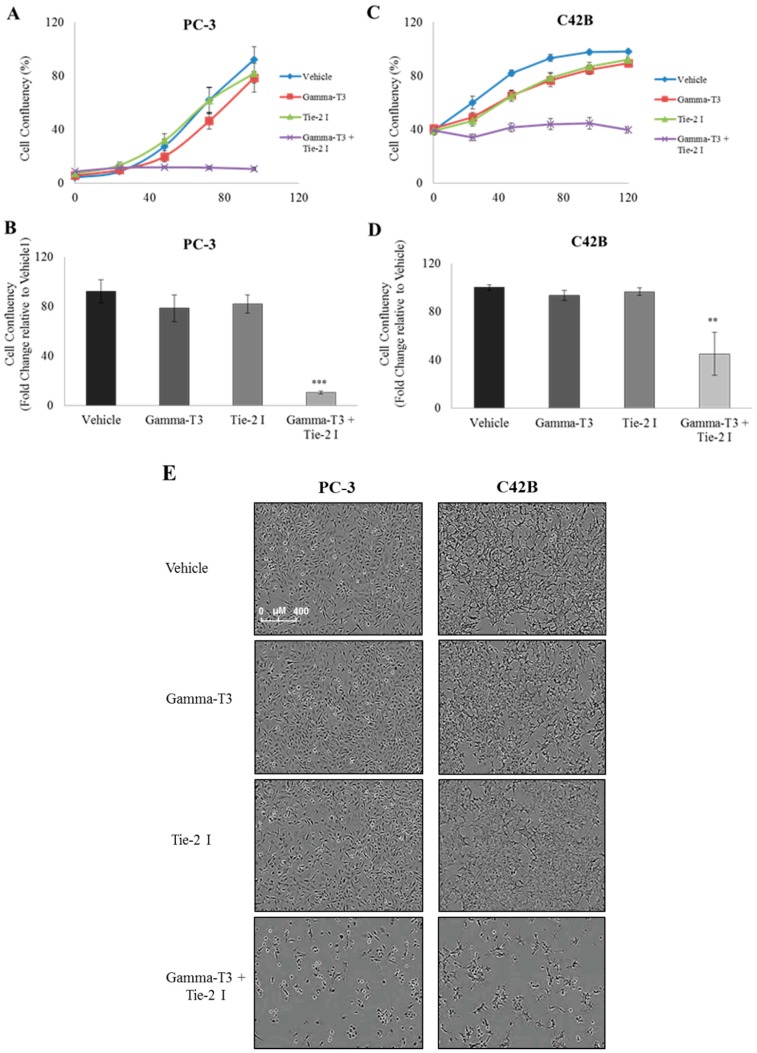
Tie-2 inhibitor promotes the anti-proliferative effect of γ-T3 against prostate cancer cells. Prostate cancer cells were treated with either a single compound (γ-T3/Tie-2 inhibitor) or a combination of both. Cell confluency (as an indicator to measure the cell growth rate) of PC-3 (**A**) and C42B (**C**) was measured by the live content cell imaging IncuCyte HD system for 96 and 120 h, respectively. Note that the combined effects of Tie-2 inhibitor and γ-T3 when used together significantly suppressed the growth rate in both PC-3 (**B**) and C42B (**D**) at both time points. Cell morphology of PC-3 and C42B (**E**) cells at 96 and 120 h after treatment is shown (10× objective). Each experiment was repeated at least three times, and the results from a representative experiment are presented as the mean ± SD. (*p* values: ** < 0.005; *** < 0.0005). Scale bar, 400 μM.

Next, we questioned whether inactivation of Tie-2 using a specific small molecule inhibitor can further enhance the anti-cancer effect of γ-T3. Both PC3 and C42B prostate cancer cells were treated with either a single compound (γ-T3/Tie-2 inhibitor) or a combination of both, and cell proliferation and viability were measured. Neither compound alone was able to induce a significant inhibitory effect on PC3 cell proliferation and viability (Figure 2A). However, when the cells were treated with both compounds, a significant reduction of cell confluency (~60%) was observed at Day 4 (Figure 2B). Examination of cell morphology confirmed that the decrease in cell confluency is due to the suppression of cell growth by the treatment (Figure 2E). Similarly, treatment of C42B cells with both compounds (10 µg/mL γ-T3 and 5 µM Tie-2 inhibitor) together, but not with either compound alone, was found to suppress cell viability (Figure 2C–E), suggesting that γ-T3 and Tie-2 inhibitor work synergistically in suppressing the survival of prostate cancer cells.

### 2.3. Inactivation of Tie-2 Enhances the Cytotoxic Effect of γ-T3

**Figure 3:**
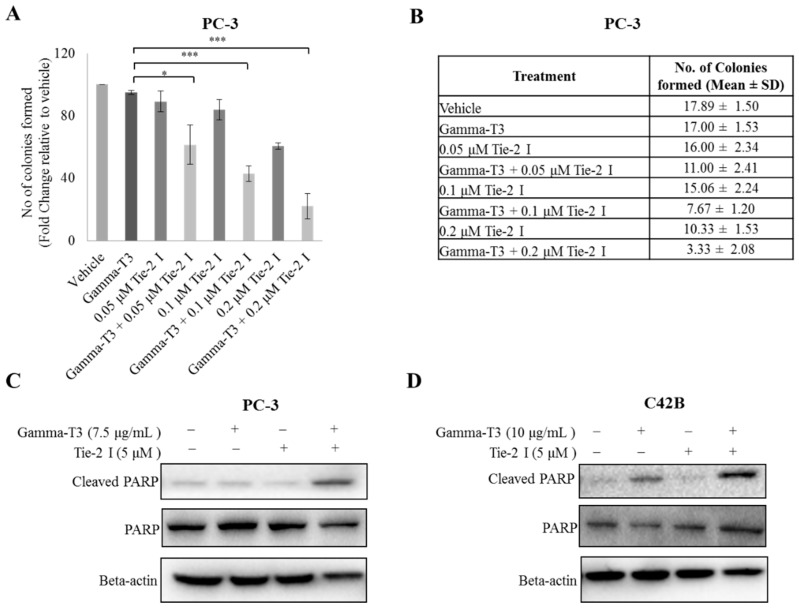
Inactivation of Tie-2 enhances the cytotoxic effect of γ-T3. PC-3 cells were treated with different combinations of Tie-2 inhibitor and γ-T3. (**A**) The number of colonies formed were stained and counted after 14 days (**A**,**B**). Protein expression of cleaved PARP, total PARP, and actin (as a loading control) in PC-3 (**C**) and C42B (**D**) cells after treating with Tie-2 inhibitor/γ-T3 or both compounds was examined by Western blotting. Each experiment was repeated at least three times, and the results are presented as the mean ± SD with representative Western blots shown. (*p* values: * <0.05; *** < 0.0005).

In order to confirm that Tie-2 inhibitor enhanced the effect of γ-T3 on prostate cancer cell viability, the colony formation assay was performed with PC-3 cells in the presence or absence of Tie-2 inhibitor and γ-T3. As shown in Figure 3A,B, at a concentration of 2 µg/mL γ-T3 had only a small effect on the colony forming ability of the cells. However, in the additional presence of Tie-2 inhibitor, the number of colonies formed was reduced significantly by approximately 40%. To further validate our findings, Western blotting analysis of the common pro-apoptotic marker PARP was performed on both PC-3 and C42B cells. The greatest upregulation of cleaved PARP was observed only in cells that were treated with both γ-T3 and Tie-2 inhibiter together (Figure 3C,D), further confirming that the inactivation of Tie-2 in prostate cancer cells enhances the cytotoxic effect of γ-T3.

### 2.4. Combination of Tie-2 Inhibitor and γ-T3 Leads to the Activation of AMPK

**Figure 4:**
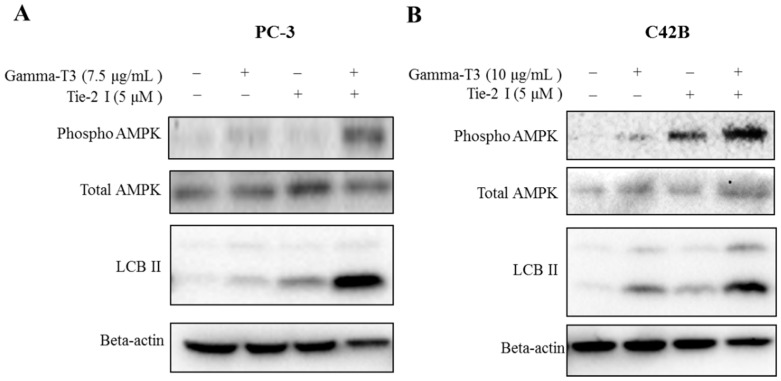
Combination of Tie-2 inhibitor and γ-T3 lead to activation of AMPK. PC-3 (**A**) and C42B (**B**) cells were treated with the indicated compounds for 48 and 72 h, respectively. Western blotting was performed to determine the level of phospho-AMPKα (Thr172), total AMPK, LCBII, and actin in both PC-3 and C42B cells. Note that the combined effect of Tie-2 inhibitor and γ-T3 led to a clear induction of phospho-AMPKα and LCBII expression in both PC-3 and C42B cells. Each experiment was repeated at least three times with representative Western blots shown.

Since activation of AMPK has recently been shown to play an important role in the anti-cancer effect of γ-T3, we examined whether Tie-2 inactivation promotes γ-T3-induced AMPK activation in prostate cancer cells. Consistent with our hypothesis, although the phosphorylation levels of AMPK at Thr172 were found to be induced by γ-T3 (7.5 μg/mL), the addition of the Tie-2 inhibitor significantly enhanced the level of AMPK phosphorylation in PC-3 cells. Interestingly, the combined treatment of γ-T3 and Tie-2 inhibitor also upregulated the protein expression of LCBII, a direct downstream target of AMPK, which plays a key role in the formation of autophagosomes during the early stage of autophagy (Figure 4A). Similar results were also observed in C42B cells, further suggesting that the combined effect of γ-T3 and Tie-2 inhibitor may induce autophagy through the activation of AMPK (Figure 4B).

### 2.5. Inhibitory Effect of Tie-2 Inhibitor and γ-T3 on Autophagic Flux

**Figure 5:**
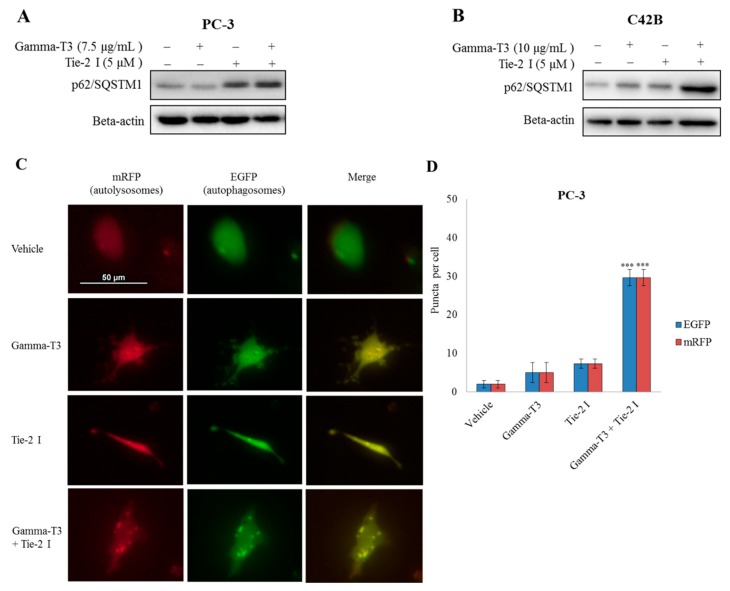
Inhibitory effect of Tie-2 inhibitor and γ-T3 on autophagic flux. Protein expression of p62 in PC-3 (**A**) and C42B (**B**) cells after treating with different combinations of Tie-2 inhibitor and γ-T3 was examined by Western blotting (representative Western blots are shown). (**C**) Measurement of autophagic flux using tandem fluorescent-tagged LC3: PC-3 cells transfected with fluorescent-tagged LC3 (mRFP-EGFP-LC3) before being treated with Tie-2 inhibitor/γ-T3 or both compounds for another 72 h (40× objective). Representative fluorescent images are shown. Red fluorescence signal represents autolysosomes (mRFP), while the green fluorescence signal represents autophagosomes (EGFP). Yellow fluorescence signal represented the merger of both mRFP and EGFP. (**D**) Cells with more than 5 puncta were quantified and at least 50 cells were counted in each group. Each experiment was repeated at least three times, and the results are presented as the mean ± SD. (*p* values: *** < 0.0005). Scale bars, 50 μM.

To determine whether the upregulation of LCBII protein after treatment with γ-T3 and Tie-2 inhibitor is a result of an induction of autophagosome formation or a blockage of lysosomal degradation of autophagosome at the later stage, Western blotting analysis of the lysosomal degradation marker, SQSTM1/p62 was performed. This combination treatment with Tie-2 inhibitor and γ-T3 was found to induce SQDTM1/p62 protein expression in both PC-3 and C42B cells (Figure 5A,B), suggesting that the induction of autophagosomes in the cells may be due to the blockage of lysosomal degradation. To further validate our findings, cells were transfected with a construct expressing a tandem fluorescent-tagged LC3 (mRFP-EGFP-LC3) before being treated with Tie-2 inhibitor and/or γ-T3. As green fluorescence (GFP) will be quenched in a low pH environment, while red fluorescence (RFP) will maintain its stability in an acidic environment, co-localisation of both fluorescent proteins (yellow) indicated that the autophagosomes do not fuse with acidic lysosomes and that the process of autophagy is therefore halted. Indeed, the treatment of cells with both Tie-2 inhibitor and γ-T3 significantly induced the number of cells that carried both the red and green punctate fluorescence signals (Figure 5C,D), supporting the notion that the induction of LCBII is due to the blockage of lysosomal degradation of the autophagosome rather than to the activation of autophagy.

## 3. Discussion

Angiogenesis is the process of new blood vessel formation. Several studies have suggested that angiogenesis is required for invasive tumour growth and metastasis [19]. Besides that, pro-angiogenic factors such as the VEGF family proteins and their corresponding receptors have been found to be highly upregulated in human cancer tissues [20]. In addition, these proteins have been found to correlate with the disease progression of numerous human cancers, including breast [21], lung [22], colorectal [23], and head and neck [24]. Recent studies have suggested that the γ-T3 was able to inhibit angiogenesis under in vitro and in vivo conditions [14,25]. In this study, we found that γ-T3 also inhibited the mRNA transcript and protein level of the pro-angiogenic factor Ang-1 in human prostate cancer cells.

Ang-1/Tie-2 is one of the well-known signalling pathways that take part in regulating angiogenesis [26]. Previous studies have suggested that prostate cancer actively secretes Ang-1, which acts in a paracrine manner to induce tumour angiogenesis during tumour metastasis and disease progression [27]. Our recent findings suggest that Ang-1/Tie-2 also functions as an autocrine loop and as a result plays an unexpected role in regulating the stemness and quiescence of prostate CSCs during the development of prostate tumour bone metastasis [18]. Therefore, the Ang-1/Tie-2 signalling pathway appears to be an ideal anti-cancer target, as its inactivation is expected to produce both direct (targeting prostate CSCs) and indirect (targeting tumour angiogenesis) anti-cancer effects. While several small molecule inhibitors are currently available for inactivating Tie-2, our finding that γ-T3 can effectively downregulate Ang-1 expression in prostate cancer cells offers an alternative approach for targeting this signalling cascade.

On the other hand, our lab also demonstrated the inhibitory effect of γ-T3 on prostate CSC self-renewal. Here, we demonstrated for the first time that the addition of exogenous Ang-1 was able to restore the CSC and quiescence markers in prostate cancer cells after treating with γ-T3, further confirming that Ang-1/Tie-2 acts as a downstream target of γ-T3. γ-T3 has been reported to have a wide range of biological actions, which include anti-diabetic, anti-inflammatory, immune-stimulatory, cardio-protective, and anti-cancer properties [28,29,30]. Accumulated evidence supports the notion that hormone-independent compared to hormone-dependent prostate and breast cancers are more responsive to γ-T3 treatment, suggesting the potential of γ-T3 in targeting hormone refractory breast and prostate cancers [10,31]. More importantly, a recent study suggested that T3 can also work synergistically with other natural anti-cancer compounds and as a result promotes their anti-cancer activity [13]. Besides that, γ-T3 has also been reported to sensitize cancer cells to chemotherapeutic drugs [32,33]. Although the exact mechanisms responsible for its broad range of biological properties is still far from clear, the AMPK signalling pathway appears to be one of the key downstream targets that mediate the anti-cancer effect of T3. Nevertheless, how T3 activates AMPK and how Tie-2 inactivation synergizes with this activation remains to be elucidated.

As a central regulator of numerous downstream targets, AMPK is known to regulate many key cellular activities important for both normal and cancer cells [34]. Autophagy, which is induced by AMPK activation, is responsible for protecting the cells from stresses such as nutrient starvation [35,36]. By inducing the degradation of excess or misfolded proteins, autophagy ensures the recycling of cellular components. Previously, γ-T3 was found to induce autophagy in cancer cells [37]. Although there was a significant upregulation of the LCBII protein level in cells treated with both γ-T3 and Tie-2 inhibitors, we found that the increase in the number of autophagosomes is not due to the induction of autophagy, but is a result of a blockage of lysosomal degradation. On the other hand, as reported by previous studies, the inhibition of autophagy can lead to energy depletion and hence contribute to cellular apoptosis [38]. This may indeed explain the effects of the combined treatment on the induction of cellular apoptosis.

## 4. Materials and Methods

### 4.1. Cell Lines and Culture Conditions

Androgen-independent prostate cancer cell lines: PC-3 cells were obtained from ATCC (Rockville, MD, USA) and were maintained in RPMI 1640 medium (Invitrogen, Carlsbad, CA, USA) supplemented with 5% fetal bovine serum (FBS, Invitrogen) and 2% (wt/vol) penicillin-streptomycin (P/S, Invitrogen). The C42B cell line was kindly provided by Dr. Leland Chung (Cedars-Sinai Medical Center, Los Angeles, CA, USA) and was maintained in T-Medium (Invitrogen) supplemented with 5% FBS and 2% P/S. All cell types were kept at 37 °C in a 5% CO_2_ environment.

### 4.2. Antibodies and Reagents

The Tie-2 inhibitor (Tie-2 I) was purchased from Santa Cruz Biotechnology, Dallas, TX, USA and was dissolved in DMSO (10 mM). Recombinant human Ang-1 recombinant protein was purchased from PROSPEC, East Brunswick, NJ, USA. Gamma-tocotrienol (γ-T3) was provided by Davos Life Science Pty Ltd. from Singapore and was dissolved in absolute ethanol (100 mM).

The following antibodies were used in this study: human CD49f, cleaved PARP, PARP, phospho-AMPK-alpha1, total AMPK-alpha1, L3BII, p62/SQSTM1, and beta-actin (Cell Signalling Technology, Danvers, MA, USA); Bmi-1 antibody (Millipore, Billerica, MA, USA); p27 antibody (BD Biosciences San Jose, CA, USA); and HRP-conjugated anti-mouse and rabbit secondary antibodies (GE Healthcare, Buckinghamshire, UK).

### 4.3. qRT-PCR Analysis

RNeasy Mini Kit (Qiagen, Germantown, MD, USA) was used to isolate the total RNA from the cells, following the manufacturer’s instructions. One microgram of RNA was used to synthesize cDNA using the SuperScript^®^ III First-Strand Synthesis Systems (Invitrogen); subsequently, qRT-PCR was carried out with the ViiA™ 7 Real-Time PCR System (Applied Biosystems, Foster City, CA, USA). ANGPT1 forward primer (5′-ACGATGGCAACTGTCGTGAG-3′) and ANGPT1 reverse primer (5′-TCCGACTTCATGTTTTCCACAA-3′) were used in this study. The transcript level of ribosomal protein L32 (*RPL32*) was used as an internal control.

### 4.4. Ang-1 ELISA

The conditioned media (CM) was collected from the cells after treatment with γ-T3 and subsequently concentrated using 10 K Amicon Ultra2-mL Centrifugal Filters (Millipore). To quantitate Ang-1 secretion by prostate cancer cells, concentrated CM was analysed using the human angiopoietin-1 DuoSet kits (R&D Systems, Minneapolis, MN, USA) following the manufacturer’s instructions and output measured using a LUMISTAR OPTIMA luminescence microplate reader.

### 4.5. Western Blot

Details regarding the experimental procedures have been described in our previous studies [39,40]. Briefly, cell pellets were lysed with lysis buffer (Cell Signalling) containing 100 µM phenylmethylsulfonyl fluoride (PMSF; Sigma-Aldrich, St. Louis, MO, USA). The cell lysates were quantitated using the Pierce™ BCA Protein Assay Kit (Thermo Fisher Scientific, Rockford, IL, USA) before loading onto a SDS-polyacrylamide gel. The resolved proteins were then transferred onto a PVDF membrane (Millipore, Billerica, MA, USA), and the membrane was subsequently probed with the indicated antibody overnight at 4 °C. The membrane was then incubated with the corresponding secondary antibodies for another hour at room temperature. After washing with TBS-T buffer, the membrane was incubated with Immobilon Western Chemiluminescent HRP Substrate (Millipore), and the signals were visualised using a Bio-Rad ChemiDoc™ XRS Gel Documentation System.

### 4.6. Cell Proliferation and Viability

Cells were counted using a Scepter^™^ Automated Cell Counter (Millipore) and seeded in 96-well plates (2 × 10^3^ per well). After 24 h, cells were then treated with Tie-2 (5 μM) inhibitor or γ-T3 (7.5 or 10 μg/mL) or both. Cell confluency (as an indicator of cell growth) was measured using a live content cell imaging IncuCyte HD system (Essen BioScience, Ann Arbor, MI, USA). Images were taken with a 10× objective at 2 h intervals for 4 (PC-3) or 5 (C42B) days. The data are presented as the percentage of cell confluency from triplicate experiments. Statistical difference was determined by a Student’s *t*-test and was considered significant if *p* <0.05.

### 4.7. Colony Formation Assay

Details regarding these experimental procedures have been described in our previous study [13]. Briefly, PC-3 cells were harvested and seeded in 12-well plate (100 cells per well). Cells were grown in the presence of γ-T3 at 2 μg/mL and/or Tie-2 inhibitor at 0.05, 0.1, or 0.2 μM. After 14 days, the colonies were stained with KaryoMAX^®^ Giemsa stain solution (Invitrogen), and the number of colonies formed was counted and normalised to that of the untreated control. Each experiment was repeated at least three times, and each data point represents the mean and standard deviation. Statistical difference was determined by a Student’s *t*-test and was considered significant if *p* <0.05.

### 4.8. Plasmid Transfection and Microscopy

Cells were transfected with 1 μg of tandem fluorescent-tagged LC3 (mRFP-EGFP-LC3, a gift from Prof Dong Yan Jin, Department of Biochemistry, The University of Hong Kong) using the FuGENE transfection reagent (Roche, Indianapolis, IN, USA) following the manufacturer’s instruction. Six hours after transfection, cells were changed to normal growth medium and treated with either 7.5 μg/mL γ-T3, 5 μM Tie-2 inhibitor, or both. After 2 days of treatment, RFP and GFP signals were determined using a Nikon fluorescence microscope. Each experiment was repeated at least three times, and each data point represents the mean and standard deviation. Statistical difference was determined by a Student’s t-test and was considered significant if *p* < 0.05.

## 5. Conclusions

Our findings suggested that the co-treatment of a Tie-2 inhibitor with γ-T3 may offer a greater opportunity in the treatment of advanced prostate cancer patients.

## Abbreviations

Ang-1	Angiopoietin-1
CM	Conditioned media
CSC	Cancer stem cell
FBS	Fetal bovine serum
γ-T3	Gamma-tocotrienol
NF-κB	Factor-kapppa B
P/S	Penicillin-streptomycin
PMSF	Phenylmethylsulfonyl fluoride
PSP	Polysaccharopeptide
RPL32	Ribosomal protein L32
TP	Tocopherol
TRF	Tocotrienol-rich fraction
